# Bridging between hype and implementation in medical extended reality

**DOI:** 10.1038/s41746-023-00972-y

**Published:** 2023-12-07

**Authors:** Oscar Freyer, Stephen Gilbert

**Affiliations:** Else Kröner Fresenius Center for Digital Health, TUD Dresden University of Technology, Dresden, Germany

**Keywords:** Health policy, Rehabilitation

## Abstract

The path to market and to a meaningful impact on care delivery for medical extended reality (MXR) is challenging, due to limitations with current display technologies and as the MXR approach is far away from the traditional practice of medicine and the daily experience of most patients or healthcare providers. Focused conferences, which bring together all stakeholders for free communication and the brainstorming of optimal approaches to design, validation, and regulatory approval are important and are being organized by the clinician-enthusiast and developer community. These conferences and the community spirit they inspire are models for other digital health subdomains.

## Introduction

There is a fraught and tortuous pathway between exciting new digital medicine concepts and their making a real and safe impact on standard care. Perhaps the degree of excitement and otherworldliness of digital technology is directly proportional to the likelihood of taking false turns and entering cul-de-sacs in development. One approach to avoiding pitfalls while bringing exciting and challenging technologies to healthcare is through focused conferences which bring together the stakeholders critical to progress. On one side are the enthusiasts, innovators, early adopters, and entrepreneurs, and on the other the regulators, implementation scientists, and healthcare providers, including those from outside the bubble of the innovation in question.

An example of this event type is the SHIFT MEDICAL (https://shiftmedical.eu) conference on MXR, which has run in Heidelberg, Germany since 2020. MXR merges the physical and digital worlds and encompasses augmented reality (AR), virtual reality (VR), and mixed reality (MR), and is certainly another universe when compared to traditional tools for medical practice. The meeting’s focus is on in-person colleague-to-colleague networking and open knowledge exchange between researchers, healthcare providers, businesses, governments, and the general public. The conference was one of the first to go “fully virtual” in 2020 but dropped this in 2022, instead adopting a fully in-person and selected attendance format. The format seems almost a reaction to the hype one might associate with VR in medicine, with instead a focus on ethical, regulatory, technological, data safety, and evidence-based principles, and is exactly what the doctor ordered for MXR.

## The application of MXR technology in medicine?

**Fig. 1:**
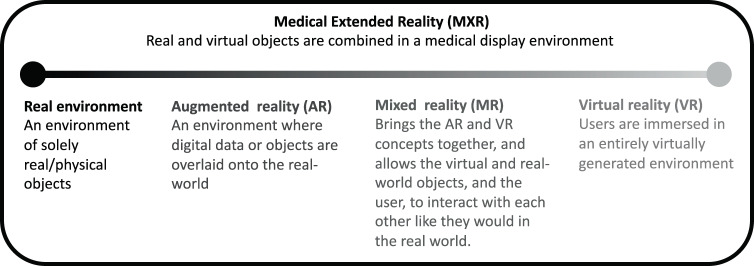
The range of MXR modalities. Definition and description of the interrelated concepts of MXR, AR, MR, and VR.

The interrelated concepts, QR, MR, and VR that make up MXR are explained in Fig. 1.

MXR approaches are deployed using a mix of head-mounted glasses, headsets, and screens. There has been a hype of the general and medical potential of these technologies in the last years, but what is their true role in medicine and how can development be better driven to meet this?

## The role of MXR in medical training

Almost all current application areas of MXR in the medical field were presented at the conference. The most advanced in implementation was the field of medical training, with products gradually being rolled out and evaluated, showing promising results in nursing education^1^ and in the training of healthcare professionals and students^2,3^. Approaches are also being researched for emergency medicine training in complex scenarios (https://cordis.europa.eu/project/id/101021775).

## The application of MXR in care

Beyond education, applications of VR were presented for aiding patient care, from cognitive rehabilitation post-stroke^4^ to physical rehabilitation^5^ and psychological therapies, e.g., to treat specific phobias, depression, and addiction^6^. Further in-development approaches on show were for VR as a tool for surgery planning^7,8^ and in radiology image viewing^9^. Some of these are already approved as medical devices in the EU and the US, a subset of which are on the market for everyday patient care. In contrast, AR can complement existing non-digitized procedures thanks to the simultaneous presentation of real-world and digital objects. There were presentations on AR approaches for precise organ transplantation through anatomical overlays and size matching in the operating room^10^ or as information-presenting interfaces on care units. Other AR applications discussed were in interventional cardiology and radiology, for assisting in planning procedures involving catheters and guiding the placement of such, with an enhancing effect on precision and efficiency^10,11^.

## The path to wider utilization

Where MXR is applied in medical devices, it is justified that it must navigate a rigorous regulatory pathway from laboratory to clinic. The main technological challenge presented was related to display technology: current flat panel displays have a physically limited depth presentation^12^. New, multifocal display technologies were described which could lead to closer-to-reality image perception, resulting in improved image quality and better depth perception^13^. Yet, the issues of headset weight, battery life, and the constraints of wired headsets remain. From a user’s perspective, poor image quality, non-ergonomic designs (e.g., weight), and unexpected or unfamiliar control conventions could limit the acceptance^14^. The FDA brought to the meeting a focus on the critical theme of usability and how this links to display and image quality^15^. The novelty of MXR technologies presents a unique set of challenges here, and new standardized evaluation methods and frameworks are needed to determine safety and effectiveness. The FDA recognizes the need for quantitative evaluation methodologies and is actively working to develop assessment methodologies they term ‘regulatory science tools.’ The collection of available regulatory science tools is freely available on the FDA’s website (https://www.fda.gov/medical-devices/science-and-research-medical-devices/catalog-regulatory-science-tools-help-assess-new-medical-devices) and can be used by both device developers and evaluators.

## A model for MXR and a model format for effective conferences

A clear message from the conference—the journey of MXR products from prototype to clinic is fraught with technical and regulatory challenges and the implementation in clinical settings is still in its early stages. These challenges are such that there should be ‘healthy skepticism’ on whether a comprehensive and widespread introduction of MXR in the healthcare sector will happen. This needs consideration of cost-effectiveness, safety, and the resolution of the known technical challenges. Standardized tests and frameworks and further development of adapted and balanced regulatory pathways will be instrumental in navigating the journey toward the clinical adoption of MXR. If you wish to get involved in the MXR movement’, can you? Although this conference is by application or invitation only this is not a barrier to those wishing to engage in MXR. This conference has a straightforward application process, and the selected group of attendees fits the concept of moving the theme forward through interdisciplinary work ‘at the coal face’ of implementation, rather than providing an audience for ‘leading experts’ to present long and largely pre-published work at a large audience.

Current and inspiring meeting designers and driven technologist-scientists could learn a lot from this format. There is much work to do at conferences like SHIFT MEDICAL, and not only for MXR but for many challenges in digital medicine and beyond.

## References

[1] Liu K, Zhang W, Li W, Wang T, Zheng Y (2023). Effectiveness of virtual reality in nursing education: a systematic review and meta-analysis. BMC Med. Educ..

[2] Lamb A, McKinney B, Frousiakis P, Diaz G, Sweet S (2023). A comparative study of traditional technique guide versus virtual reality in orthopedic trauma training. Adv. Med. Educ. Pract..

[3] Mergen M (2023). Immersive training of clinical decision making with AI driven virtual patients - a new VR platform called medical tr.AI.ning. GMS J. Med. Educ..

[4] Specht J, Stegmann B, Gross H, Krakow K (2023). Cognitive training with head-mounted display virtual reality in neurorehabilitation: pilot randomized controlled trial. JMIR Serious Games.

[5] Lazem H (2023). The extent of evidence supporting the effectiveness of extended reality telerehabilitation on different qualitative and quantitative outcomes in stroke survivors: a systematic review. Int. J. Environ. Res. Public Health.

[6] Wiebe A (2022). Virtual reality in the diagnostic and therapy for mental disorders: a systematic review. Clin. Psychol. Rev..

[7] Hattab G (2021). Investigating the utility of VR for spatial understanding in surgical planning: evaluation of head-mounted to desktop display. Sci. Rep..

[8] Sibrina D, Bethapudi S, Koulieris GA (2023). OrthopedVR: clinical assessment and pre-operative planning of paediatric patients with lower limb rotational abnormalities in virtual reality. Vis. Comput..

[9] Uppot RN (2019). Implementing virtual and augmented reality tools for radiology education and training, communication, and clinical care. Radiology.

[10] Stephenson N (2023). Extended reality for procedural planning and guidance in structural heart disease – a review of the state-of-the-art. Int. J. Cardiovasc. Imaging.

[11] Li Y (2018). A wearable mixed-reality holographic computer for guiding external ventricular drain insertion at the bedside. J. Neurosurg..

[12] Reichelt, S., Häussler, R., Fütterer, G. & Leister, N. Depth cues in human visual perception and their realization in 3D displays. *In*: Three-Dimensional Imaging, Visualization, and Display 2010 and Display Technologies and Applications for Defense, Security, and Avionics IV 7690:92–103 (SPIE, 2010).

[13] Zhan T, Xiong J, Zou J, Wu S-T (2020). Multifocal displays: review and prospect. PhotoniX.

[14] Souchet AD, Lourdeaux D, Pagani A, Rebenitsch L (2023). A narrative review of immersive virtual reality’s ergonomics and risks at the workplace: cybersickness, visual fatigue, muscular fatigue, acute stress, and mental overload. Virtual Real..

[15] Beams R (2022). Evaluation challenges for the application of extended reality devices in medicine. J. Digit. Imaging.

